# Monoclonal Gammopathy of Undetermined Significance Diagnosed by Persistent Anemia Following Living Kidney Transplantation: A Case Report

**DOI:** 10.7759/cureus.81183

**Published:** 2025-03-25

**Authors:** Mitsuru Tomizawa, Shunta Hori, Kuniaki Inoue, Tatsuo Yoneda, Kiyohide Fujimoto

**Affiliations:** 1 Department of Urology, Nara Medical University, Kashihara, JPN

**Keywords:** anemia, erythropoiesis-stimulating agent, kidney transplantation, monoclonal gammopathy of undetermined significance, renal anemia

## Abstract

The prevalence of monoclonal gammopathy of undetermined significance increases with age, and there may be undiagnosed patients among kidney transplant recipients. A 69-year-old woman underwent ABO-incompatible living-donor kidney transplantation. Her renal function improved immediately postoperatively. Her hemoglobin level gradually increased to 10.5 g/dL at 3 months postoperatively but gradually decreased to 7.3 g/dL at 9 months postoperatively. We administered an erythropoiesis-stimulating agent and consulted the Department of Hematology. Bone marrow aspiration revealed that the pathological monoclonal plasma cells constituted 4.6% of bone marrow cells. Immunoelectrophoresis identified serum monoclonal proteins as immunoglobulin G lambda. The patient was diagnosed with monoclonal gammopathy of undetermined significance, and free light-chain measurements were performed regularly. No disease progression was observed. The anemia improved with the administration of an erythropoiesis-stimulating agent. Monoclonal gammopathy of undetermined significance should be explored as a cause of persistent anemia after kidney transplantation.

## Introduction

Kidney transplantation (KT) is considered the best treatment for improving the quality of life and prognosis in patients with end-stage renal disease (ESRD), including the growing older population in Japan [1,2]. Monoclonal gammopathy of undetermined significance (MGUS) is a plasma cell disorder defined by a serum monoclonal protein <3 g/dL, bone marrow plasma cell infiltration <10%, and, most importantly, the absence of end-organ damage. The prevalence of MGUS in the general population is 3.2%, 5.3%, and 7.5% in those aged ≥50 years, ≥75 years, and ≥85 years, respectively; thus, the prevalence increases with age [3]. Although the underlying mechanism is unknown, MGUS reportedly occurs more frequently in patients with chronic kidney disease [4-6], and there may be undiagnosed patients with MGUS among KT recipient candidates. MGUS is considered a precancerous condition, and the risk of progression from MGUS to multiple myeloma or related disorders is approximately 1% per year [3]. After KT, permanent oral immunosuppressant administration is essential to suppress rejection. KT is performed for patients with ESRD and MGUS, but immunosuppressants may promote malignant transformation and infection; therefore, regular screening is recommended [7].

We report on a case of a patient undergoing living-donor KT who had not been tested for MGUS preoperatively and was diagnosed with MGUS owing to persistent postoperative anemia. The Institutional Review Board for Clinical Studies (Medical Ethics Committee ID: 2749) at Nara Medical University determined that ethical review was not required for this study. Consent to participate and for publication was acquired from the patient.

## Case presentation

**Figure 1:**
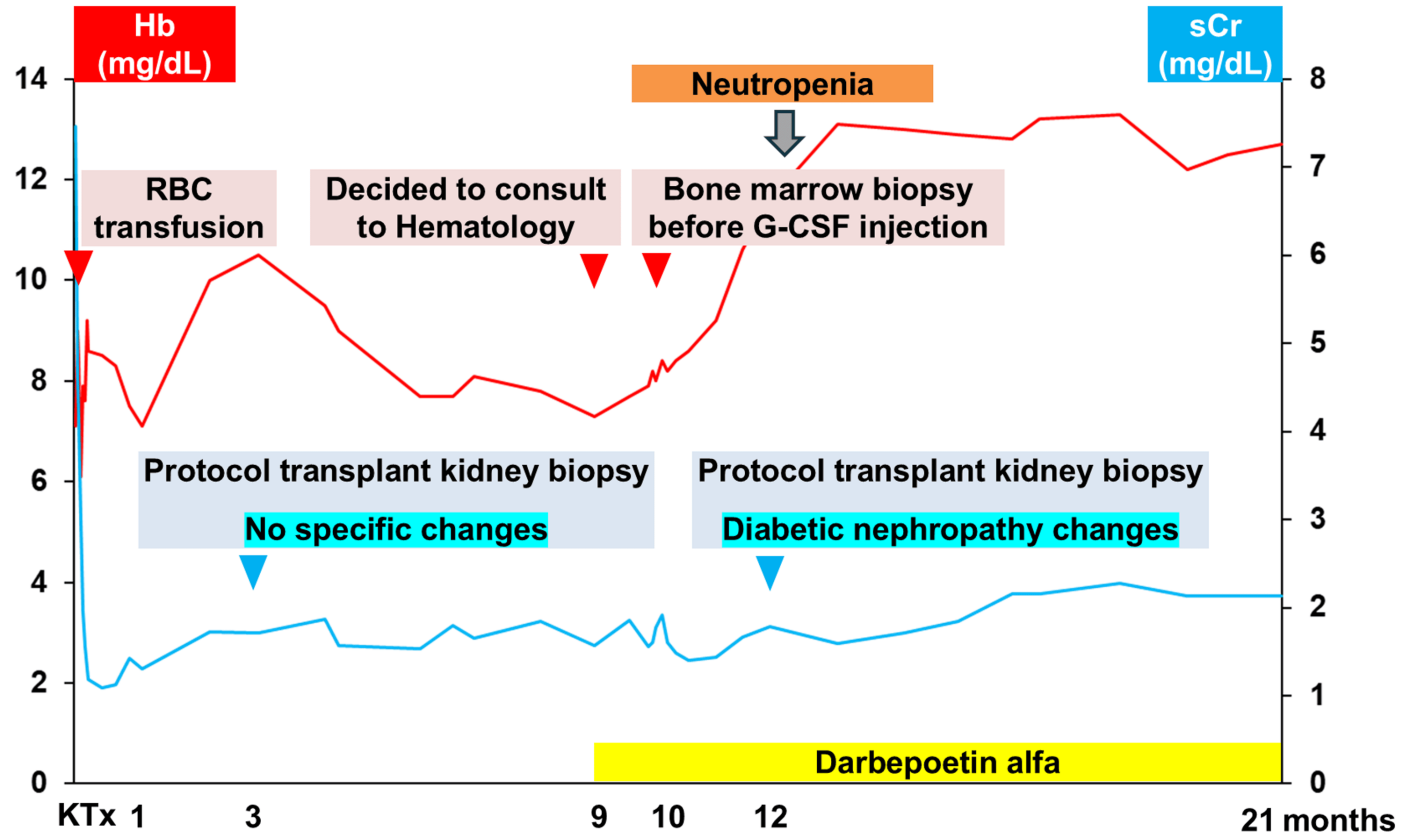
Clinical course of the patient Changes in post-transplant hemoglobin and serum creatinine levels are seen. The hemoglobin level gradually increased from one to three months postoperatively, gradually decreasing thereafter. Hb, hemoglobin; sCr, serum creatinine; RBC, red blood cells; G-CSF, granulocyte-colony stimulating factor; KT, kidney transplantation; MGUS, monoclonal gammopathy of undetermined significance

**Figure 2:**
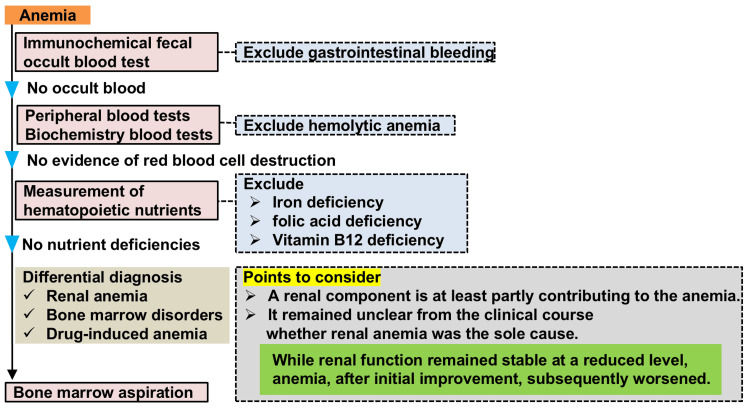
Differential diagnostic flowchart for anemia in this patient Blood tests and immunological fecal occult blood tests ruled out gastrointestinal bleeding, hemolytic anemia, and deficiencies in hematopoietic nutrients. Although renal factors partially contributed to the anemia, the clinical course made it unclear whether renal anemia was the sole cause. Therefore, bone marrow aspiration was performed.

**Figure 3:**
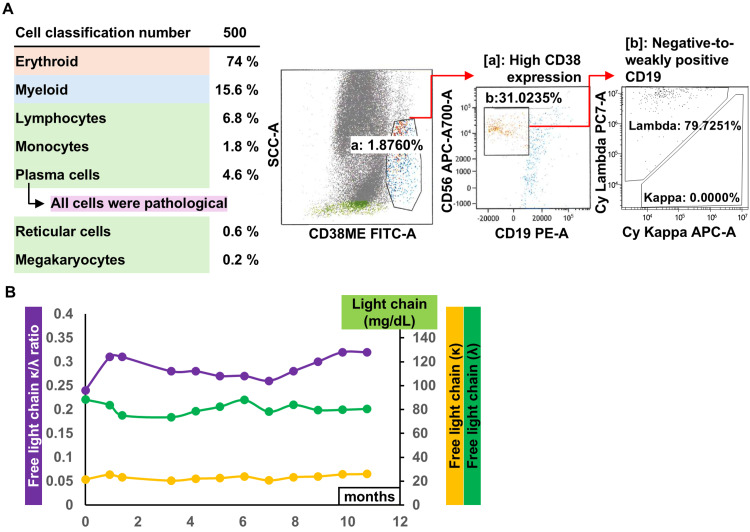
Bone marrow aspiration findings and changes in the free light chain (A) Bone marrow aspiration findings. Pathological plasma cells constituted 4.6% of bone marrow cells, and flow cytometry revealed a bias toward lambda-type cells with (a) high CD38 expression and (b) negative-to-weakly positive CD19. (B) Changes in the free light chain after diagnosis of MGUS. There has been no progression of the disease for 11 months.

A 69-year-old woman underwent ABO-incompatible living-donor KT with her husband as the donor. She had started hemodialysis owing to ESRD secondary to diabetic nephropathy eight months preoperatively. After starting hemodialysis, an erythropoiesis-stimulating agent (ESA) was administered to treat renal anemia and was discontinued after KT. As a desensitization therapy before KT, rituximab was administered, and double filtration plasmapheresis and plasma exchange were performed. The initial immunosuppressants were tacrolimus, mycophenolate mofetil, prednisolone, and basiliximab, and this was kept as maintenance immunosuppression except for basiliximab. All these protocols were performed according to our institutional protocol. Famotidine was administered for gastric mucosal protection, and sulfamethoxazole-trimethoprim was administered to prevent pneumocystis pneumonia. Her renal function improved immediately postoperatively, and she experienced no surgical complications. Post-transplant hemoglobin and serum creatinine levels are shown in Figure 1. Four units of red blood cells were transfused postoperatively. One month postoperatively, she developed cytomegalovirus viremia, which resolved after two weeks of treatment with oral valganciclovir. The hemoglobin level gradually increased to 10.5 g/dL from 1 to 3 months postoperatively but gradually decreased to 7.3 g/dL at 9 months postoperatively despite the absence of iron deficiency or gastrointestinal bleeding. We referred her to the Department of Hematology on her subsequent visit one month later, and ESA (darbepoetin alfa) administration was resumed. In the Department of Hematology, her blood count showed neutropenia, which was considered drug-related. Laboratory results at the first visit to the hematology department are shown in Table 1. Figure 2 shows the differential diagnostic flowchart for anemia in this patient, along with key considerations. Although erythropoietin levels were elevated, the increase was insufficient, suggesting that a renal component was at least partially contributing to the anemia. However, while renal function remained stable at a reduced level, anemia, after initial improvement, subsequently worsened. Therefore, the clinical course did not allow for the conclusion that renal anemia was the sole cause. The mycophenolate mofetil dose was reduced, and sulfamethoxazole-trimethoprim was discontinued. Before the granulocyte-colony stimulating factor (G-CSF) injection, a bone marrow aspiration was performed by the Department of Hematology because G-CSF can modify bone marrow aspiration results, preventing an accurate diagnosis. After the bone marrow aspiration, G-CSF was administered. Bone marrow aspiration revealed pathological plasma cells constituting 4.6% of bone marrow cells, and flow cytometry revealed a bias toward lambda-type cells with high CD38 expression and negative to weakly positive CD19 (Figure 3A). The patient had anemia, which required differentiation from multiple myeloma but was diagnosed with MGUS for several reasons: (1) no progression on regular free light chain measurements (Figure 3B); (2) protocol transplant kidney biopsy performed one year after KT showed no pathological changes owing to light chain deposition; and (3) anemia was improved by ESA administration. At 11 months after diagnosis, MGUS had not progressed.

**Table 1:** The laboratory results at the first visit to the hematology department

Variable		Measured	Normal range
White blood cell count	/ µL	1000	3300-8600
Stab cell	%	3.0	0.5-6.5
Segmented cell	%	28.0	38.0-74.0
Lymphocyte	%	33.0	16.5-49.5
Monocyte	%	27.0	2.0-10.0
Eosinophil	%	9.0	0.0-8.5
Basophil	%	0.0	0.0-2.5
Atypical lymphocyte	%	0.0	0.0
Hemoglobin	g/dL	7.9	11.6-14.8
Hematocrit	%	25.1	35.1-44.4
Mean corpuscular volume	fL	94.0	83.6-98.2
Reticulocyte	%	3.4	0.8-2.2
Platelet count	10^4^/µL	20.0	15.8-34.8
Serum creatinine level	mg/dL	1.56	0.46-0.79
Estimated glomerular filtration rate	mL/min/1.73m^2^	26.0	60.0-120.0
Serum blood urea nitrogen	mg/dL	26	8-20
Serum sodium level	mg/dL	141	138-145
Serum potassium level	mg/dL	4.7	3.6-4.8
Serum chloride level	mg/dL	112	101-108
Serum calcium level (corrected)	mg/dL	9.7	8.8-10.1
Serum phosphorous level	mg/dL	3.1	2.7-4.6
Total bilirubin	mg/dL	0.5	0.4-1.5
Serum albumin level	mg/dL	3.5	4.1-5.1
Tacrolimus concentration (trough)	ng/mL	3.4	Target; around 5
Erythropoietin level	mIU/mL	26.6	4.2-23.7
Vitamin B12 level	pg/mL	986	233-914
Folic acid level	µg/dL	3.2	2.4-10.0
Serum iron	µg/dL	44	33-179
Total iron-binding capacity	µg/dL	247	263-457
Transferrin saturation	%	18	More than 20%
Ferritin	ng/mL	516	12-152

## Discussion

We demonstrated that MGUS should be considered a possible cause of persistent anemia after KT. In this case, the anemia improved rapidly with the use of ESAs, and MGUS may have been masked by general renal anemia treatment.

The incidence of post-transplant anemia is 20-50% [8]; most cases improve soon after transplantation [9]. The following factors can cause early post-transplant anemia: (1) iron deficiency, (2) intraoperative blood loss and postoperative blood sampling, (3) immunosuppressant use, and (4) infections [9]. In this case, the anemia improved until the third month postoperatively, but it progressed again thereafter; therefore, we suspected a bone marrow disease. Diagnostic criteria for MGUS include the absence of anemia and renal failure; however, in clinical practice, patients with MGUS often have both anemia and chronic kidney disease [4,10,11], making the diagnosis difficult. A study examining the patterns of multimorbidity in MGUS patients and the general population reported that anemia (MGUS patients: 43%; controls: 16%) and CKD (MGUS patients: 36%; controls: 18%) were more common among MGUS patients [11]. In multiple myeloma that develops as MGUS progresses, anemia is observed in 70% of patients and is the most common clinical symptom [12]. The pathophysiological mechanisms of multiple myeloma-related anemia involve plasma cell-produced cytokines that inhibit erythropoiesis and impair iron homeostasis [12]. If chronic kidney disease coexists with MGUS, the symptoms of renal anemia may be more pronounced. Further research on the relationship between anemia and chronic kidney disease in patients with MGUS is needed. Furthermore, despite the diagnostic criteria for MGUS, the impact of macroglobulinemia with respect to anemia and renal failure must be considered on an individual basis in kidney transplant recipients and candidates. In this case, we carefully considered whether the patient had MGUS or clinically significant macroglobulinemia and whether treatment was appropriate, and we decided to observe the patient's condition. Diagnosing MGUS and determining the appropriate timing for therapeutic intervention in this population need to be considered based on transplant kidney biopsy and changes over time in free light chains.

For patients with post-transplant anemia, iron supplementation is given for iron deficiency; otherwise, ESA or hypoxia-inducible factor prolyl-hydroxylase inhibitors are used to treat renal anemia [9], which may occur in KT recipients with functional single kidneys and chronic kidney disease that persists post-surgery. In this case, anemia improved promptly after the use of ESA. Therefore, this case suggests that if persistent post-transplant anemia is observed, MGUS should be considered as a differential diagnosis before using ESA.

Currently, there are no clear guidelines on the management of KT recipients with MGUS or MGUS occurring after KT. Several studies have demonstrated that KT recipients with MGUS have a low rate of malignant transformation, even in the long term, and that MGUS does not adversely affect graft survival or overall survival [7,13,14]. In the present case, MGUS was not evaluated preoperatively. However, even if MGUS had been diagnosed before transplantation, KT would have proceeded, given the low lifetime risk of MGUS progression following KT. A case report showed that KT for patients with MGUS may improve patient well-being and satisfaction with daily social and physical activities, compared to the pre-KT levels [15]. MGUS that develops after KT may have a different pathology from MGUS present before KT [13]. Specifically, post-transplant MGUS is often associated with cytomegalovirus infection or human herpesvirus type 8 infection; approximately one-third of cases are transient, and it generally has a better prognosis than pre-existing MGUS [13]. Although reducing the dose of immunosuppressants is not recommended, regular surveillance is recommended as immunosuppressant use may promote malignant transformation regardless of post-transplant or pre-existing MGUS [7]. The diagnosis of MGUS in patients undergoing KT is important for appropriate surveillance, and persistent anemia after KT may suggest the diagnosis. Moreover, because of the relatively high prevalence of MGUS, some reports recommend preoperative screening of KT candidates with a history of plasma cell disorder, age >50 years, or unexplained nephrotic-range proteinuria during kidney disease [7]. Further evidence is required to inform the preoperative screening of MGUS.

## Conclusions

Persistent anemia after kidney transplantation, particularly when it worsens without progressive deterioration of renal function over time, may be a sign of monoclonal gammopathy of undetermined significance. It should be considered when using erythropoiesis-stimulating agents. The diagnosis of potential monoclonal gammopathy of undetermined significance may be useful for proper follow-up after kidney transplantation.
